# Histiocytic sarcoma; case report of a rare disease in a kidney transplant recipient

**DOI:** 10.12860/jnp.2015.18

**Published:** 2015-07-01

**Authors:** Pedro Ventura Aguiar, Carla Dias, Pedro Azevedo, Hugo Neves Silva, Manuela Almeida, Sofia Pedroso, La Salete Martins, Leonídio Dias, Anabela Rodrigues, Ramón Viscaíño, António Cabrita, António Castro Henriques

**Affiliations:** ^1^Renal Transplant Unit, Nephrology Department, Centro Hospitalar do Porto, Portugal; ^2^Department of Pathology, Centro Hospitalar do Porto, Portugal

**Keywords:** Histiocytic sarcoma, Post-transplant lymphoproliferative disease, Malignancy, Transplant, Chronic kidney disease

## Abstract

*Background:* Histiocytic sarcoma (HS) is a rare hematologic neoplasm with a few hundred cases having been described to date.

*Case Presentation:* We report the case of a 56-year-old woman with a history of hepatitis C infection and chronic kidney disease (CKD), submitted to a kidney transplant in 1984, under maintenance immunosuppression with prednisone and azathioprine. Patient presented with a relentlessly growing mass on her right front thorax. It was painless, smooth, and adherent to the deep muscle. Laboratory studies were unremarkable. Ultrasonography and computerized tomography (CT) scan revealed a highly vascularized heterogeneous mass (8×9 cm), with a necrotic centre. Positron emission tomography (PET) scan demonstrated multiple thoracic, abdominal, and pelvic nodules. Histology revealed a highly undifferentiated HS (vimentin, CD68, CD99, and CD4 positive). In spite of having started treatment with etoposide and thalidomide, no clinical response was achieved and the patient died three months later.

*Conclusions:* To the authors’ knowledge, this is the first described case of HS in a solid organ transplant patient.

**Implication for health policy/practice/research/medical education:**

A World Health Organization (WHO) expert panel stated histiocytic sarcoma is likely to be underdiagnosed. The case herein presented provides evidence to the existence of this rare disease in solid organ transplant recipients. Therefore this diagnosis should be considered in the presence of an undifferentiated sarcoma (vimentin and CD68 positive). Moreover, the presented case highlights the importance of positron emission tomography (PET) scan for the diagnosis of disseminated disease.

## 1. Introduction

Post-transplant lymphoproliferative disease (PTLD) incidence among solid organ recipients ranges from 0.56%-1.8% (1-3), and is a frequent cause of death with functioning graft (US Renal Data System 2013 Annual Data Report).

Histiocytic sarcoma (HS) is a rare hematologic neoplastic disease with only a few hundred cases reported worldwide, with a wide range of clinical presentations and manifestations described (4-7). This heterogeneity of reports unsuits analysis for a potential standard of care for either diagnosis or treatment.

Herein we present a report of a case an HS in a kidney transplant recipient. To the best of authors’ knowledge, this is the first report of an HS in a solid organ recipient not related to Epstein-Barr virus (EBV) infection.

## 2. Case Presentation

A 56-year-old woman with a history of chronic hepatitis C virus (HCV) infection diagnosed with chronic kidney disease (CKD) of unknown aetiology at the age of 27 years, was submitted to a deceased-donor kidney transplant in 1984, and maintained under immunosuppression with prednisone and azathioprine. Post-transplant period was complicated with new-onset diabetes after transplantation (NODAT), and noninvasive cutaneous squamous cell carcinoma diagnosed in 2004 – surgically removed and without evidence of relapse. The remaining transplant period was unremarkable with a serum creatinine of 0.8 mg/dL in December 2011.

**Figure 1:**
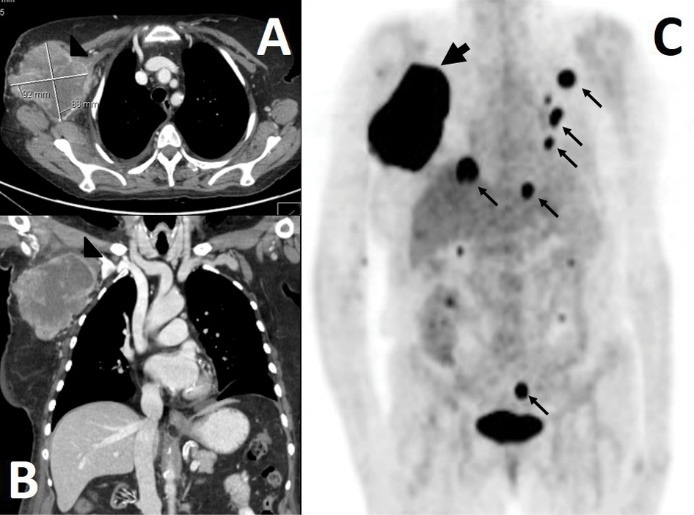
Figures A (transversal) and B (coronal) represent CT scan images of the subclavian lesion at admission (arrowhead). Figure C represents a whole body capture of the PET scan, with marking the subclavian primary lesion (large arrow) and metastatic lesions in thorax, abdomen and pelvis (small arrows).

Patient presented in 2012 to the emergency department due to fever of unknown origin. She also complained of a painless, smooth, and adherent to the deep muscle relentlessly growing mass on her right front thorax. Physical exploration was otherwise unremarkable – no other masses, palpable lymph nodes, or splenomegaly were found. Normal leucocyte count and haemoglobin levels were found, as were lactic dehydrogenase (LDH) and bilirubin levels. EBV serology was positive for IgG but negative for IgM. Both ultrasound and computerized tomography (CT) scan (Figure 1A and B) revealed a highly vascularized heterogeneous mass (8×9 cm), with a necrotic centre. All other imaging studies – bone scintigraphy, mammography, and mammary ultrasonography  –revealed only small residual nodules on the thorax, without evidence of brain, abdominal or pelvic masses. Immunophenotyping of both peripheral blood and bone marrow was unremarkable. Blood cultures were positive for methicillin-sensitive *Staphylococcus aureus,* successfully treated with cephazolin.

**Figure 2:**
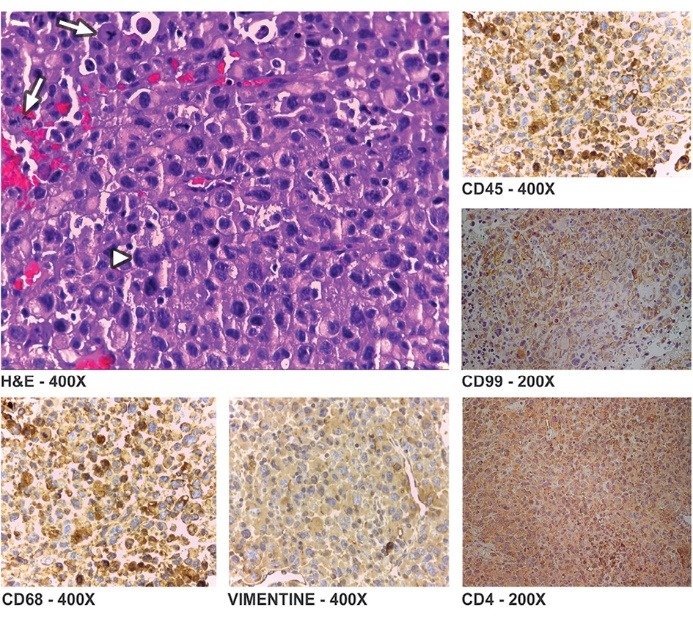
Histological analysis of the five specimens, between 4 and 5mm, revealed a malignant undifferentiated large cell neoplasm composed of sheets of large, round to oval cells, in an inflammatory background. Neoplastic cells had abundant eosinophilic cytoplasm sometimes with a xanthomatous appearance, large pleomorphic nuclei with fine chromatin and prominent nucleoli. Focal emperipolesis (arrowhead) and frequent mitotic figures (arrows) were also seen.

Histology of the biopsied lesion revealed a malignant undifferentiated large cell neoplasm. No lymphoid tissue was identified. The immunohistochemical study was positive for vimentin, CD68, CD99, and CD4, and negative for all other tumour markers (Figure 2), consistent with the diagnosis of HS (Table 1).

**Table 1:** Description of immunohistochemical markers of different solid and myeloproliferative tumors, and results found favoring the diagnosis of histiocytic sarcoma, or monocytic/myeloid sarcoma

**Differential diagnosis**	**Immunohistochemical marker**	**Result**
Soft tissue sarcoma	Vimentine;	Positive
	Desmin; Alpha-actinMyo-Dl; CD34	Negative
Metastatic melanoma	S100; HMB45: Melan A; CD117	Negative
Undifferentiated carcinoma	AE 1/AE3; CAM5.2; EMA	Negative
Alk(+) anaplastic large cell lymphoma	ALK; C30; EMA; T-cell markers; Lysozyme	Negative
T-cell lymphoma	CD20; CD 19; CD79a	Negative
Monocytic/ Myeloid Sarcoma	CD4	Positive
	CD45	(+/-)
	CD43	(+/-)
	Lysozyme	Negative
	Myeloperoxidase	Negative
	CD117	Negative
	CD68	Positive
	CD163	Not available
	Vimentine	Positive
Histiocytic sarcoma	CD45	(+/-)
	CD43	(+/-)
	CD68	Positive
	CD163	Not available
	CD4	Positive
	Lysozyme	Negative
	Vimentine	Positive
Langerhans cell histiocytosis/sarcoma	S100; CD1a	Negative
Classical Hodking lymphoma	CD30; CD15	Negative

A positron emission tomography (PET) scan, later performed, revealed a large axillary mass, as well as multiple thoracic, abdominal, and pelvic nodules (Figure 1CC). Evidence of disseminated disease precluded treatment with systemic chemotherapy. Due to failure to prevent the mass growth, thalidomide was added to etoposide after 1 month. Patient eventually died 3 months after the diagnosis.

## 3. Discussion

The cumulative incidence of PTLD has been reported to approach 1.8% (1–3), and represents 16% of all deaths from malignancy among solid organ recipients (8).

HS is a rare neoplastic disease of unknown aetiology, accounting for less than 1% of all hematolymphoid neoplasms (9). It most often affects males (59.4%), with a bimodal age distribution; a smaller peak between 0-29 years, and a larger one between 50-69 years old (9). Clinical presentation varies from focal lesions to disseminated disease (7), but most frequently affects the gastrointestinal tract, skin, and lymph nodes. Surgical removal with adjunctive chemotherapy and/or radiotherapy may be curative for focal disease (5). For disseminated HS, different chemotherapy schemes with etoposide, thalidomide, and stem cell transplantation have been used, with variable outcomes (5-7). Nevertheless, prognosis is still limited, with most patients dying within 2 years after the diagnosis.

Previously known as “true histiocytic lymphoma,” HS is a diagnosis of exclusion. The classification according to the 2008 World Health Organization (WHO) requires the verification of the histiocytic lineage and the exclusion of other, poorly differentiated, large-cell malignancies. Even though, the presence of B-cell markers no longer excludes its diagnosis (10).

HS immunohistochemical profile most often expresses lysosome-associated (macrophage or histiocytic) markers, CD68 (in 100% of cases) and lysozyme (in 94% of cases) (11). Additionally, a negative staining is expected for molecules related to B-cells, T-cells, accessory or dendritic cells, myeloid cells, epithelial cells, and melanocytes. Because of the monocytic origin of histiocytes, monoblastic leukaemia should be excluded (12). Recently, CD163, a haemoglobin scavenger receptor, has been recognized as a new macrophage-related differentiation marker, which is more specific than the conventional histiocyte-related molecules (8).

Steroids, a cornerstone of immunosuppression in solid organ transplantation, inhibit gene induction by blocking the translocation of nuclear factor-κB (NFκB) from cytoplasm to nucleus. As a result, gene transcription and release of inflammatory cytokines is impaired (13). Azathioprine, a purine analogue derivative of 6-mercaptopurine, is a broad myelocyte suppressant. It incorporates into cellular DNA and inhibits gene replication and the consequent T-cell activation (13).

Immunosuppression vintage was over 25 years in the presented case. To the authors’ comprehension, the prolonged gene transcription inhibition due to long-term treatment with steroids enhanced the risk for mutation in the B and/or T-cells, possibly causing a differentiation into histiocytes or macrophages. Moreover, the association with azathioprine could have enhanced the risk for translational mutations and proliferation of a mutated monoclonal clone. Nevertheless, a large number of kidney transplant recipients are under an immunosuppression scheme similar to the one reported, and still the incidence of HS is very low.

To the best of authors’ knowledge, this is the first report of HS in a solid organ recipient without evidence of EBV infection. In 1985, Kramer et al (14) described an EBV infection associated with HS, one year post kidney transplant. However, definition, etiology, and diagnosis of this entity has considerably changed ever since, with the advent of novel immunohistochemistry of B-cell lineage findings (10). Moreover, presently there is compelling evidence for a lack of relationship between EBV infection and an increased the risk for HS (9).

## 4. Conclusions

This report highlights two important features: first, the importance of PET scan for the diagnosis of disseminated disease. Neither CT scan nor bone scintigraphy were able to identify the presence of thoracic, abdominal, or pelvic metastasis. Second, the need for increased awareness for HS, since an overconfident diagnosis of the other more frequent neoplasms likely results in a reduced recognition of HS’s true incidence.

## Authors’ contribution

PVA conducted manuscript redaction, CD, histological diagnosis, and picture selection. PA; Prepared draft. HNS; Patient management. MA; Patient management. SP; Manuscript revision. LSM; Manuscript revision and patient management. LD; Manuscript revision. AR; Patient follow-up. RV; Histological diagnosis, and pathology report. AC; Manuscript revision. ACH; Manuscript revision.

## Conflicts of interest

None of the contributing authors have any conflict of interest, including specific financial interests or relationships and affiliations relevant to the subject matter or materials discussed in the manuscript.

## Funding/Support

None.
